# Septum pyramidal adjustment and repositioning – a conservative and effective rhinoplasty technique^☆^

**DOI:** 10.1016/j.bjorl.2017.11.009

**Published:** 2017-12-26

**Authors:** Nedio Atolini, Vanessa Lunelli, Gustavo Pereira Lang, Luís Fernando Melotti, Tatiany Tiemi Yamamoto, Emílio Jonatas Muneroli

**Affiliations:** a Hospital São Vicente de Paulo, Departamento de Otorrinolaringologia, Passo Fundo, RS, Brasil; b Universidade Federal da Fronteira Sul (UFFS), Hospital São Vicente de Paulo, Passo Fundo, RS, Brasil

**Keywords:** Rhinoplasty, Nasal dorsum, Nasal pyramid, Nasal function, Rinoplastia, Dorso nasal, Pirâmide nasal, Função nasal

## Abstract

**Introduction:**

In rhinoplasty, the nasal dorsum has important relevance regarding the esthetic and functional aspects of the surgery. Its reduction should be performed with maximum accuracy and controlled resection to prevent or minimize potential complications. The septum pyramidal adjustment and repositioning consists of a conservative surgical technique of the nasal dorsum, which does not require the detachment of the upper lateral cartilages of the nasal septum, allowing the remodeling of the nasal dorsum while maintaining esthetic lines and nasal function, potentially reducing frequent complications in more traditional surgeries.

**Objective:**

To describe the septum pyramidal adjustment technique in detail, presenting its advantages and disadvantages in relation to the other surgical approaches, as well as to disclose results of this surgical procedure in patients submitted to primary rhinoplasty in a specific hospital.

**Methods:**

The medical records of all patients submitted to surgery from 2011 to 2015 through this surgical technique were evaluated by the same team. Of these cases, certain variables were analyzed such as gender, age, indication for reoperation and surgical complications.

**Results:**

153 patients underwent rhinoplasty through septum pyramidal adjustment. Of these, 13 patients experienced an indication for a second surgery and four had some type of postoperative complication.

**Conclusion:**

The septum pyramidal adjustment surgical technique is a simple procedure, as it does not require the reconstruction of the nasal dorsum. It has a low number of complications and preserves the anatomical structures.

## Resumo

**Introdução:**

Na rinoplastia, o dorso nasal tem importante relevância no quadro estético e funcional. A sua redução deve ser realizada com máxima precisão e ressecção controlada a fim de prevenir ou minimizar complicações potenciais. O termo *septum pyramidal adjustment and repositioning* consiste em uma técnica cirúrgica conservadora do dorso nasal, que não requer a desinserção das cartilagens laterais superiores do septo nasal, que permite remodelar o dorso nasal, manter as linhas estéticas e a função nasal, reduz potencialmente complicações frequentes nas cirurgias mais tradicionais.

**Objetivo:**

Descrever em detalhes o *septum pyramidal adjustment*, expor suas vantagens e desvantagens em relação às outras abordagens cirúrgicas, bem como apresentar resultados desse procedimento cirúrgico em pacientes submetidos à rinoplastia primária em um hospital específico.

**Método:**

Foram avaliados os registros médicos de todos os pacientes operados de 2011 a 2015 por essa técnica cirúrgica, pela mesma equipe. Desses casos, foram analisadas algumas variáveis, como: sexo, idade, indicação de reoperação e complicações cirúrgicas.

**Resultados:**

Foram submetidos 153 pacientes à rinoplastia por *septum pyramidal adjustment*. Desses, 13  tiveram segunda indicação cirúrgica e quatro apresentaram alguma complicação pós-operatória.

**Conclusão:**

A técnica cirúrgica *septum pyramidal adjustmen* apresenta-se como um procedimento de fácil execução, pois não exige a reconstrução do dorso nasal. Apresenta baixo número de complicações e preserva as estruturas anatômicas.

## Introduction

Rhinoplasty is one of the most complex surgical procedures, one that requires precise results, in which the borderline between a favorable outcome and error is quite tenuous. Moreover, the dynamics observed between different nasal areas, as well as the variety of available surgical techniques and esthetic goals make this procedure very challenging.^1^ Thus, nasal dorsum reduction is one of the main targets of rhinoplasty and an essential step for surgeons who want to achieve the desired results.

During the nasal dorsum approach, aiming to prevent or minimize potential complications, it is extremely important for the surgeon to have comprehensive knowledge of the nasal dorsum anatomy and its relevance regarding the esthetic and functional aspects.^2^ The nasal skeleton consists of the posterior septum and nasal bones, in its cephalic portion, and in the upper lateral cartilages and the cartilaginous septum in its caudal portion. During the rhinoplasty, the nasal dorsum reduction refers to the reduction of its individual components (septum, cartilage, bone and mucosa), with maximum precision and controlled resection.^3^

Traditionally, the nasal dorsum is modified to correspond to the nasal tip esthetics.^2^ Reduction of the dorsal hump, when performed without an adequate evaluation, can lead to unfavorable cosmetic results and compromise the nasal function. Among the potential consequences are included dorsum irregularities, nasal valve narrowing, inverted “V” deformity, and over or under-resection of the dorsum.

The acronym S.P.A.R. (Septum Pyramidal Adjustment and Repositioning), consists in a conservative surgical technique of the nasal dorsum that does not require the detachment of the upper lateral cartilages of the nasal septum, in contrast to what occurs in the classic rhinoplasty.^4^ This surgical procedure allows the remodeling of the nasal dorsum, while maintaining esthetic lines and nasal function, potentially reducing frequent complications in more traditional surgeries.

The aim of this study is to describe the S.P.A.R. technique in detail, while presenting its advantages and disadvantages in relation to the other surgical approaches, as well as to present the results of this surgical procedure in patients submitted to primary rhinoplasty in Service of Otorhinolaryngology and Cervicofacial Surgery of the Hospital São Vicente de Paulo, Passo Fundo, RS.

## Methods

This study was based on a sample of patients from the public health system and a private clinic. All patients included in this study underwent S.P.A.R., a technique considered by this team as the first option for most of the primary rhinoplasties in the nasal dorsum approach.

The authors reviewed several nasal dorsum approach methods in the literature, and found few articles describing the technique.

The medical records of all 153 patients submitted to surgical procedures from 2011 to 2015 utilizing this surgical technique were evaluated by the same team. Of these cases, certain variables were analyzed such as gender, age, indication for reoperation and surgical complications.

The study protocol was approved by the Research Ethics Committee (approval n. 55143216.0.0000.5564).

### The surgical technique

**Figure 1:**
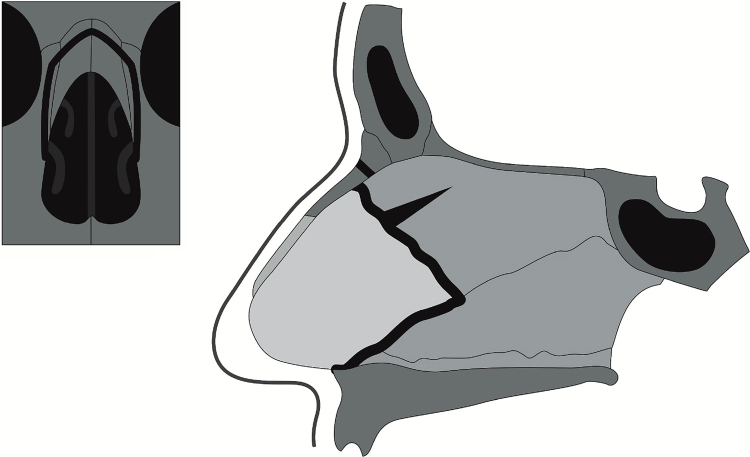
Schematic representation of the S.P.A.R. surgical technique.

The S.P.A.R. is based on the technique performed by Wilson Dewes^4^ during his decades of facial plastic surgery practice. Dewes, influenced by the technique first described by Cottle,^5^, ^6^ adapted the initial knowledge to the results obtained during his activity in private practice. The schematic representation of the surgical technique is shown in Fig. 1.

The surgery can be performed under sedation or general anesthesia, depending on the surgeon and surgical team experience. If the surgeon chooses sedation, the infraorbital and nasociliary nerves should be blocked with a solution of lidocaine, bupivacaine and epinephrine. Subsequently, the septum, nasal dorsum, nasal tip and lateral and transverse fracture areas, are infiltrated with the same solution, respecting the maximum dose of each component.

**Step 1**: After a 10-minute interval to attain the appropriate vasoconstriction effect, the surgery begins with a hemitransfixion or transcolumellar septal incision. Next access to the nasal tip, the septum and the nasal dorsum structures is achieved.

**Step 2**: Once the incision is made, the surgeon proceeds to detach the four septal tunnels to have access to all the osteocartilaginous regions.

**Figure 2:**
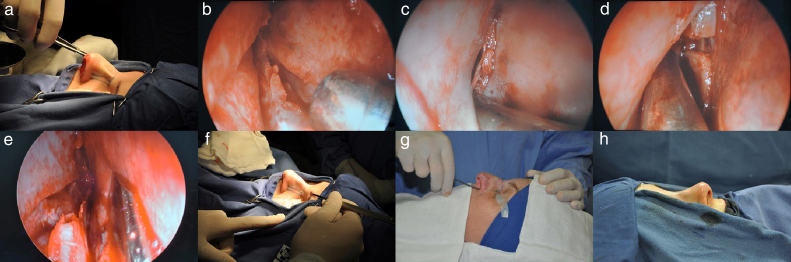
Schematic steps. (A) Nasal dorsum dissection. (B) Inferior chondrotomy. (C) Posterior chondrotomy. (D) Removal of part of the perpendicular lamina of the ethmoid. (E) Removal of the maxillary crest. (F) Transverse fracture of the nasal bone. (G) Lateral fracture of the nasal bone. (H) Postoperative outcome.

**Step 3**: After getting access to the septal tunnels, the nasal dorsum detachment is performed; this can be achieved through open or closed approach, depending on the surgeon's choice in each case, as shown in Fig. 2A. After that, the dorsal hump can be rasped without opening the nasal dorsum, that is, a delicate rasping to correct minor irregularities.

**Step 4**: A small portion of the perpendicular lamina of the ethmoid must be removed along its length, as shown in Fig. 2B–D. It is sometimes necessary to remove a small vertical strip of cartilage in the posterior portion of the quadrangular cartilage, to decrease the tension after the “push down maneuver”.^4^ In cases where the perpendicular lamina of the ethmoid and the vomer show deviations, they can be removed without impairing the final esthetic results.

**Step 5**: Part of the maxillary crest is removed – in cases with deviation – or a strip of the horizontal septal cartilage in the region where it articulates with the maxillary crest throughout the length from the anterior nasal spine, as shown in Fig. 2E. It is important to clarify that the osteocartilaginous parts can be removed *en block* – if both have a deviation – paying attention to remove a sufficient amount to lower the dorsum and with no fluctuation.

**Step 6**: Transverse fractures ahead of the dorsal hump. For these fractures, transcutaneous access may be chosen medially to the eyebrow with a 2- or 3-mm thick angled osteotome, as shown in Fig. 2F.

**Step 7**: After that, the surgeon should perform lateral fractures, as shown in Fig. 2G. In cases where the protuberance is quite prominent, double lateral osteotomies should be considered. Asymmetrical fractures can also be carried out in cases of a deviated noses.

**Step 8**: After the entire nose is mobilized, it is possible to push down the nasal dorsum (“push-down” maneuver). The movement should be smooth, and, in case of resistance, one should be aware of possible incomplete fractures, insufficient removal of the osteocartilaginous structures or incomplete removal of the posterior cartilaginous septal, as shown in Fig. 2H.

**Step 9**: Some adjustments may be necessary, such as light rasping of the dorsum and placement of the cartilage graft at the radix level to camouflage some residual protuberance, Fig. 2H.

Because the technique proposes the reduction of nasal height through an “*en block*” lowering, accessory maneuvers can be critical to reach the final goal. Therefore, a turbinoplasty increases the area of the internal nasal valve and reduces the chance of nasal obstruction, since the push-down maneuver reduces the internal nasal area.

Also, nasal lowering sometimes induces a downward rotation of the nasal tip and can also make it prominent in height. Thus, the techniques for nasal tip rotation and height reduction may be necessary.

Finally, nasal vestibule enlargement and increase of the nasal base are frequent in some cases; thus, nostril reduction softens the contour lines of the nasal base.

As in all rhinoplasties, it is essential to securely attach the septum to the anterior nasal spine. The authors prefer to use 4-0 monocryl to suture the septum and internal incisions; and perform skin sutures with 6-0 mononylon. Transseptal sutures are preferable to nasal splints.

Nasal dressings should be used, while exercising light pressure to keep the dorsum in the ideal position.

## Results

Of the 153 patients submitted to surgery, 55 (35.9%) were men and 98 (64.1%) were women. The mean age was 27.2 years.

Of all 153 cases, 13 patients had a second surgical indication to correct a residual hump and, in 3 patients, a third surgery was performed to correct nasal dorsum irregularities.

The S.P.A.R. technique usually has a low rate of surgical complications. Of the above mentioned cases, 3 patients had nasal bleeding in the early postoperative period and required nasal packing for one day; 6 patients, in the period from 2011 to 2014, during the intraoperative period, had excessive rasping of the nasal dorsum, which was opened, and had to be reconstructed with spreader grafts, and therefore were excluded from the series. Additionally, one patient had a septal hematoma and had to undergo surgical drainage.

**Figure 3:**
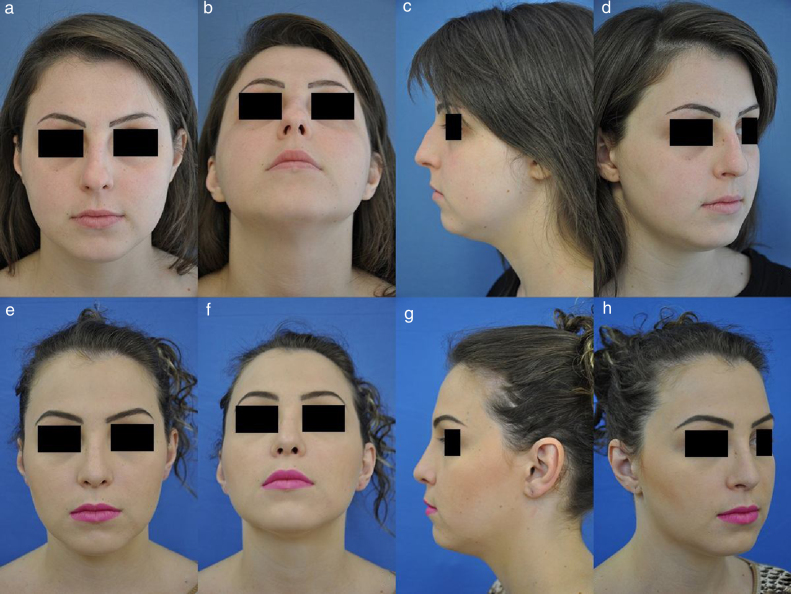
Case 1 (A–D) preoperative appearance of a patient with dorsal hump and a boxy tip. (E–H) Patient's postoperative appearance 7 months after being submitted to rhinoplasty. S.P.A.R. technique, cartilage graft in the radix, nasal tip strut and alar turn-in flap.

**Figure 4:**
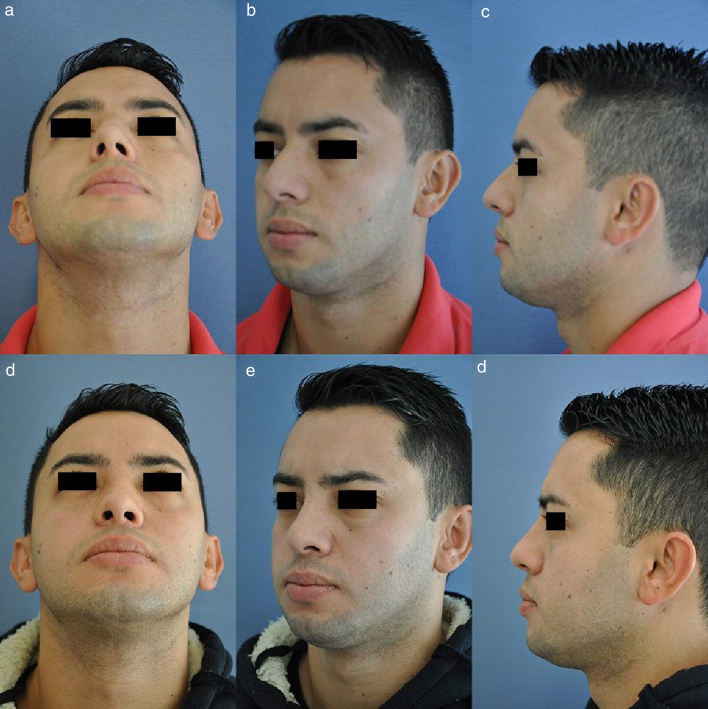
Case 2 (A–C) preoperative aspect of a patient with dorsal hump. (D–F) Postoperative appearance of the patient one year after being submitted to the S.P.A.R. technique.

Figure 3, Figure 4 illustrate the result of the S.P.A.R. in 2 patients. Case 1 shows the preoperative appearance and after 7 months of surgery, while the second case shows the appearance before surgery and one year after the procedure.

## Discussion

The excessive nasal hump is one of the main complaints of patients undergoing esthetic rhinoplasty. Thus, adequate reduction of the nasal dorsum is a determinant factor for surgical success and, consequently, for patient satisfaction. The favorable appearance of the nasal dorsum depends on the correct recreation and/or preservation of the so-called dorsal esthetic lines, which determine the esthetic frontal view of the dorsum.^1^

The nasal dorsum is formed by an osteocartilaginous component, which consists at its base of the nasal bones and distally by the upper lateral cartilages and septum. The formation of nasal prominence in most patients depends mainly on the cartilaginous portion, of which characteristics vary according to the strength and thickness of the cartilages, differing according to gender, age, ethnicity and history of trauma (accidental or iatrogenic).^7^

The earliest descriptions of rhinoplasty date from the late nineteenth century. However, it was only in 1931, with the systematization developed by Joseph, that rhinoplasty was popularized throughout the world.^8^ It is known that Rubin's osteotome was, for many years, the classic tool to approach the nasal hump, leading to high impact osteotomies without direct visualization, increasing the risk of hypo/hyper-resections and asymmetric alterations in the dorsal lines.^9^ Recently, the use of rasping was introduced and allowed a more precise reduction and modeling of the bone with less trauma.

The complications associated with traditional approaches to the nasal dorsum, include dorsal irregularities, narrowing of the nasal valve, inverted “V” deformities, etc. It is known that the best strategy for the correction of a prominent nasal dorsum is to avoid excessive resection, considering that high-impact osteotomies (often observed in classical rhinoplasties) may lead to the collapse of the K (Keystone) area, formed by the confluence of the nasal bones, perpendicular lamina of the ethmoid, upper lateral cartilage and septal cartilage, representing the basic and fundamental structure of the nasal pyramid. Additionally, weakening of the nasal structure may result in the collapse of the internal nasal valve, leading to nasal obstruction and physiological breathing alterations.^5^

Thus, some reconstruction techniques have been described to minimize such complications. The dorsal spreader grafts, obtained from septal cartilage, consist of cartilaginous plates of 5–6 mm in height and 30–32 mm in length, fixed between the septum and the upper lateral cartilages. In primary rhinoplasty, the main objectives of using these grafts, of which obtaining, and fixation require considerable technical capacity of the surgeon, is the recreation of the nasal dorsum while maintaining the dorsal esthetic lines. Additionally, they work on nasal physiology, by increasing the angle of the internal nasal valve, thus reducing the risk of obstruction.

The spreader flaps technique proposes folding the upper lateral cartilages and suturing them to the septum, as done with the spreader grafts. As an advantage, this technique is faster and easier to perform. However, another important point of this technique is related to the fact that the cartilage flap is available near the area to be reconstructed. However, as with the spreader grafts, the use of spreader flaps may lead to a widening of the nasal dorsum, affecting the dorsal esthetics, especially in patients who already have a wide nasal pyramid.^9^

The use of grafts in the nasal dorsum is still aimed to camouflage dorsal irregularities. Aiming to improve dorsal esthetic lines, several materials and techniques have been described in the literature. Examples include the use of autologous cartilage (usually nasal septum), bone (collected from the nasal septum, mastoid, lower turbinate and even the tibia and olecranon), soft tissues (cadaveric dermis, autologous fat and fibrous tissue), synthetic implants (using materials such as silicone and polyethylene) and osteocartilaginous glue.^10^, ^11^

In this sense, the septum pyramidal adjustment and repositioning has shown to be a less traumatic technique, since its main objective is to preserve the structures of the nasal dorsum, while reducing the nasal pyramid height.^12^, ^13^

## Conclusion

The S.P.A.R. surgical technique is an easier procedure to perform when compared to the traditional rhinoplasty techniques, mainly because it does not require the nasal dorsum reconstruction. In addition to the technical feasibility, this surgical procedure reduces the number of complications, is less traumatic and preserves the anatomical structures.

The authors consider S.P.A.R. a good surgical option for patients with high radix, narrow nasal dorsum, with no intense irregularities, thin skin, osteocartilaginous hump and without severe nasal and septal deviations.

## Conflicts of interest

The authors declare no conflicts of interest.
